# Supplementary material for: Mathematical Models of Topically and Intravitreally Applied Ranibizumab
Source: Invest Ophthalmol Vis Sci. 2025 Aug 20;66(11):45. doi: 10.1167/iovs.66.11.45 (PMC12372946; doi:10.1167/iovs.66.11.45)
Supplement: Supplement 1 [file iovs-66-11-45_s001.pdf]

Mathematical models of topically and intravitreally applied ranibizumab

IOVS — Supplementary Material

Paul A. Roberts*[1,2], Chloe N. Thomas[3], Gabriel Bellamy Plaice[3], James A. Roberts[3], Marie-Christine Jones[4], James W. Andrews[5] and Lisa J. Hill[3,6]

[1]Centre for Systems Modelling and Quantitative Biomedicine, University of Birmingham, Birmingham, United Kingdom

[2]Department of Optometry and Visual Sciences, City St George's, University of London, London, United Kingdom

[3]Department of Biomedical Sciences, University of Birmingham, Birmingham, United Kingdom

[4]School of Pharmacy, University of Birmingham, Birmingham, United Kingdom

[5]School of Mathematics, University of Birmingham, Birmingham, United Kingdom

[6]Birmingham Biomedical Research Centre, National Institute for Health and Care Research, University Hospitals Birmingham, Birmingham, United Kingdom

*Corresponding author

E-mail address: p.a.roberts@univ.oxon.org (Paul A. Roberts)

Markov Chain Monte Carlo — further details

Uniform priors were chosen to span two orders of magnitude above and below the original fitted values, with $\beta_{\mathrm{Tear-Aq},r} \in [6\times10^{-9}, 6\times10^{-5}]$ and $\beta_{\mathrm{Aq-Vit},r} \in [0.01, 100]$ for model fit 1 and $\beta_{\mathrm{Aq-Vit},r} \in [6\times10^{-3}, 60]$ for model fit 2. The target distribution was sampled in $\log_{10}$ space to prevent over sampling from higher magnitude parameter values. A Gaussian transition kernel was used for sampling (in $\log_{10}$ space), with the mean taking the value of the previous step and a standard deviation of $\sigma_k = 0.02$ for both parameters in model fit 1, and $\sigma_k = 0.1$ for model fit 2. The noise was assumed to have a Gaussian distribution, with standard deviation $\sigma = 10^{-3}$ in model fit 1 and standard deviation $\sigma = 0.1$ for model fit 2 at all data points (ensuring an even weighting of each data point). The values of σ_k and σ were chosen, in part, to obtain acceptance rates close to the often quoted optimum of 0.234,[1] achieving an acceptance rate of 0.254 for model fit 1 and of 0.250 for model fit 2.The total number of MCMC iterations was 3×10^6 for both model fits. Initial parameter values were chosen as the original fitted values ($(\beta_{\mathrm{Tear-Aq},r}, \beta_{\mathrm{Aq-Vit},r}) = (5.93\times10^{-7}, 0.929)$ for model fit 1 and $\beta_{\mathrm{Aq-Vit},r} = 0.577$ for model fit 2), avoiding the need for a burn in. Alternative initial values were also tested, with the chains converging to the same region of parameter space.

References

1. A. Gelman, W. R. Gilks, and G. O. Roberts. Weak convergence and optimal scaling of random walk Metropolis algorithms. *Ann. Appl. Probab.*, 7(1):110–120, 1997.

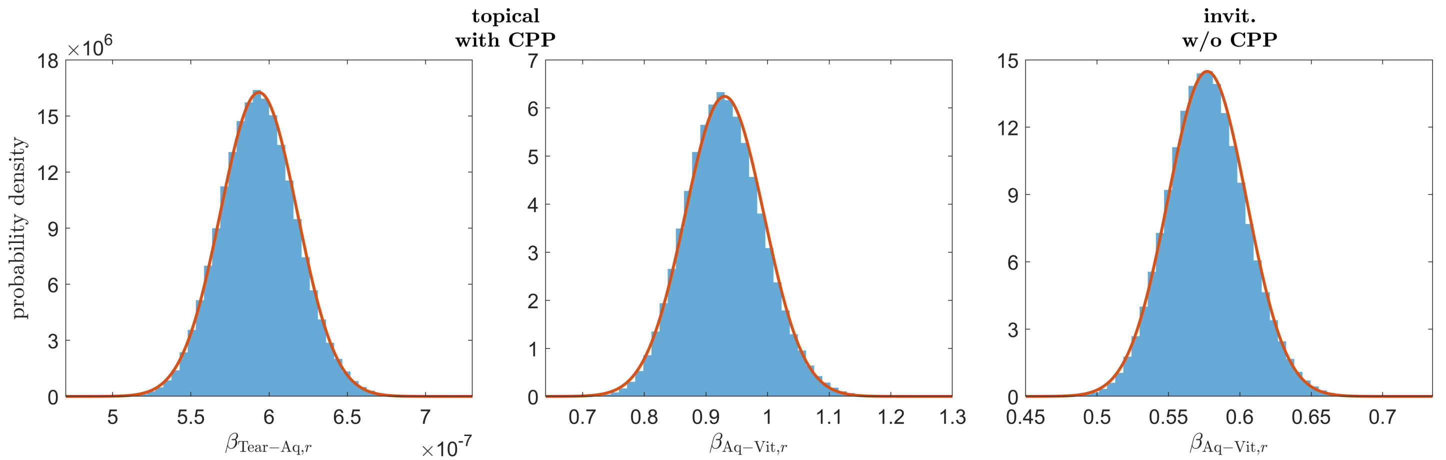

Figure S1: Posterior distributions for MCMC fits to *ex vivo* porcine data. Panels show posterior distributions for $\beta_{\mathrm{Tear-Aq},r}$ and $\beta_{\mathrm{Aq-Vit},r}$ under topical drop administration with CPP (**left and centre panels**) and for $\beta_{\mathrm{Aq-Vit},r}$ under intravitreal injection without CPP (**right panel**). **Left and centre**: a single drop is applied at $t = 0$ hr; simulations start at $t = 0$ hr with $r_{\mathrm{Tear}}(0) = r_{\mathrm{Dose}} = 2.07 \times 10^4$ pmol ml^{-1}, $r_{\mathrm{Aq}}(0) = 0$ pmol ml^{-1} and $r_{\mathrm{Vit}}(0) = 0$ pmol ml^{-1}; fitting was performed for r_{Aq} at $t = 20$ min, 40 min and 3.5 hr to the mean data points; Equations 3–13 were solved with depleting tear ranibizumab concentration, constant tear volume and in the absence of VEGF. **Right**: a single injection is administered at $t = 0$ hr; simulations start at $t = 20$ min ($= 1/3$ hr) with $r_{\mathrm{Aq}}(1/3) = 4.73 \times 10^{-2}$ pmol ml^{-1} and $r_{\mathrm{Vit}}(1/3) = 4.29$ pmol ml^{-1}, equal to the mean data points at those times; fitting was performed for r_{Aq} at $t = 40$ min, 1 hr and 3.5 hr to the mean data points; Equations 4–12 were solved in the absence of VEGF. See ‘Markov Chain Monte Carlo — further details’ in the Supplementary Materials for further information on the MCMC implementation. The posterior distributions show a single sharp peak for each parameter in all cases. All remaining parameters chosen as the default porcine values in Table 3.

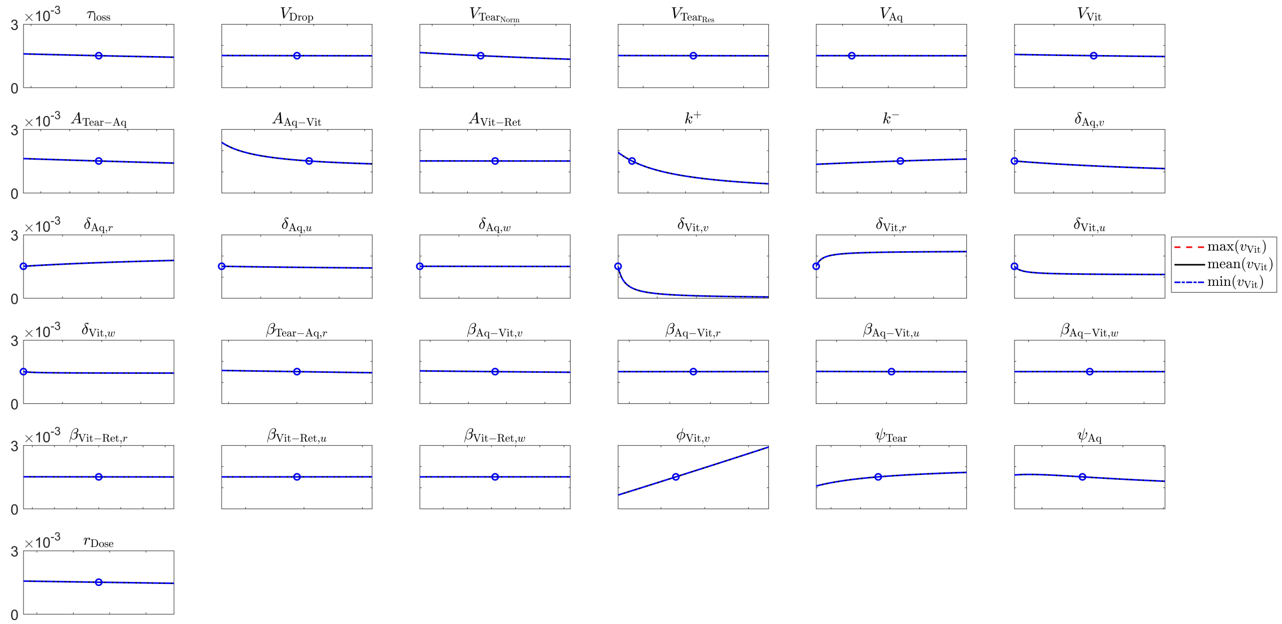

Figure S2: Sensitivity of vitreal VEGF concentration to parameter variation, with topical drop therapy. Panels show variation in the maximum/mean/minimum vitreal VEGF (V) concentration, in response to variation in model parameters over biologically realistic ranges. Equations 2–12 were solved for $t \in [0, 12]$ weeks, with topical drops applied on the hour, every hour. Parameters were varied individually, across 101 values uniformly distributed over the ranges given in Table 4, the remaining parameters being held at their default values given in Table 4. For each parameter set, the maximum/mean/minimum vitreal values of V were calculated over model outputs from the interval $t \in [9, 12]$ weeks. Circles show the maximum/mean/minimum values of V for the default parameter set (see Table 4).

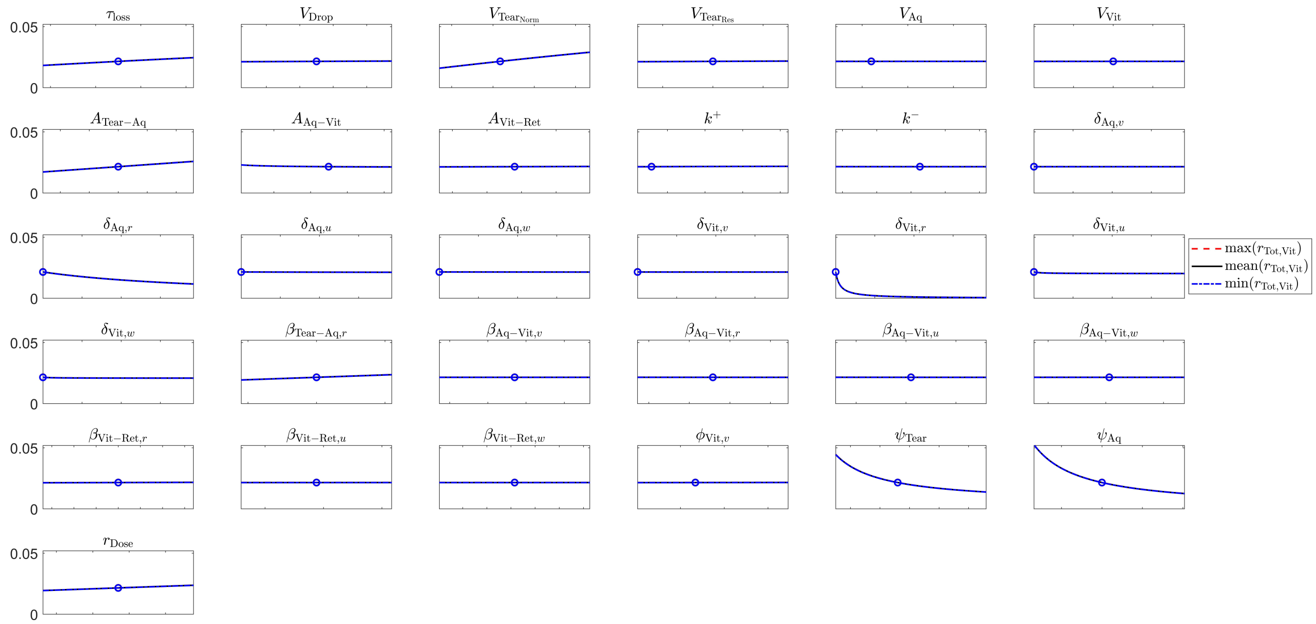

Figure S3: Sensitivity of total vitreal ranibizumab concentration to parameter variation, with topical drop therapy. Panels show variation in the maximum/mean/minimum total vitreal ranibizumab (R_{Tot} = R + VR + 2RVR) concentration, in response to variation in model parameters over biologically realistic ranges. Equations 2–12 were solved for $t \in [0, 12]$ weeks, with topical drops applied on the hour, every hour. Parameters were varied individually, across 101 values uniformly distributed over the ranges given in Table 4, the remaining parameters being held at their default values given in Table 4. For each parameter set, the maximum/mean/minimum vitreal values of R_{Tot} were calculated over model outputs from the interval $t \in [9, 12]$ weeks. Circles show the maximum/mean/minimum values of R_{Tot} for the default parameter set (see Table 4).

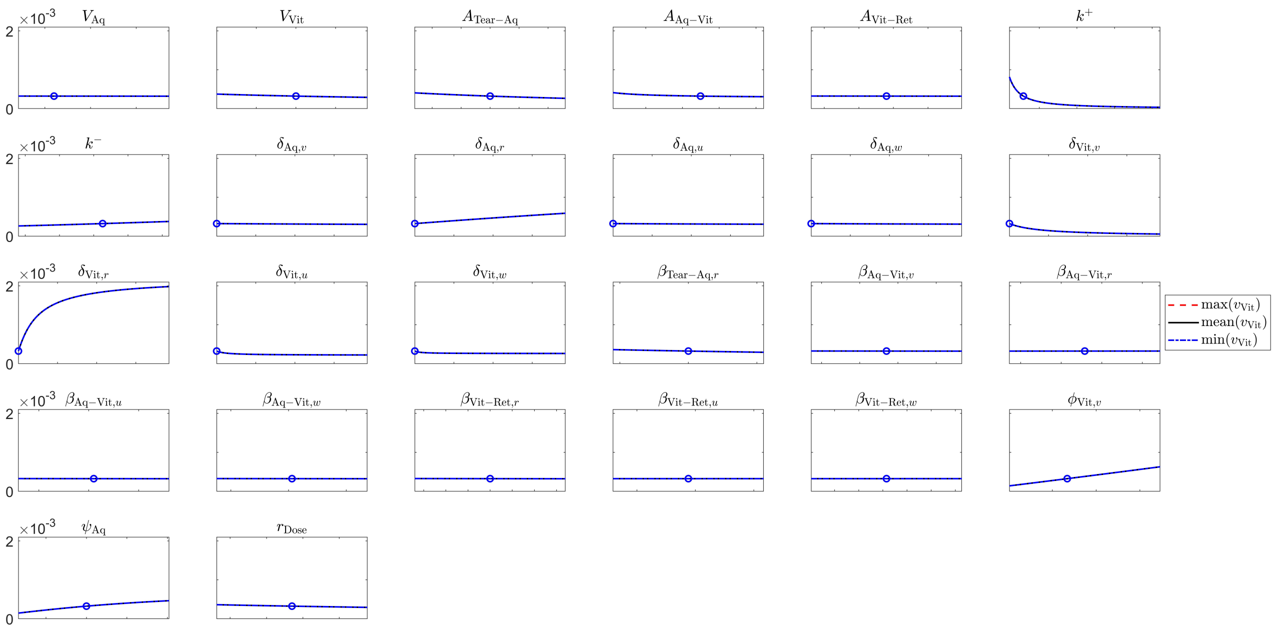

Figure S4: Sensitivity of vitreal VEGF concentration to parameter variation, with drug-eluting contact lens therapy. Panels show variation in the maximum/mean/minimum vitreal VEGF (V) concentration, in response to variation in model parameters over biologically realistic ranges. Equations 2–12 were solved for $t \in [0, 12]$ weeks, with a drug-eluting contact lens, worn continuously. Parameters were varied individually, across 101 values uniformly distributed over the ranges given in Table 4, the remaining parameters being held at their default values given in Table 4. For each parameter set, the maximum/mean/minimum vitreal values of V were calculated over model outputs from the interval $t \in [9, 12]$ weeks. Circles show the maximum/mean/minimum values of V for the default parameter set (see Table 4).

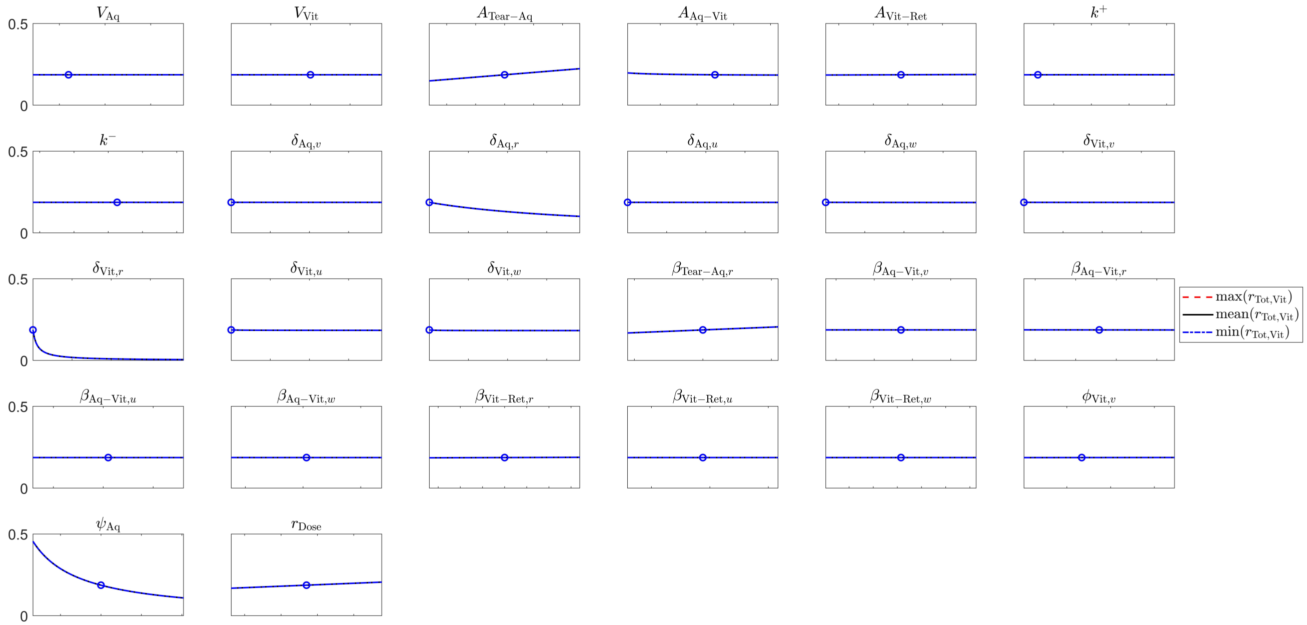

Figure S5: Sensitivity of total vitreal ranibizumab concentration to parameter variation, with drug-eluting contact lens therapy. Panels show variation in the maximum/mean/minimum total vitreal ranibizumab (R_{Tot} = R + VR + 2RVR) concentration, in response to variation in model parameters over biologically realistic ranges. Equations 2–12 were solved for $t \in [0,12]$ weeks, with a drug-eluting contact lens, worn continuously. Parameters were varied individually, across 101 values uniformly distributed over the ranges given in Table 4, the remaining parameters being held at their default values given in Table 4. For each parameter set, the maximum/mean/minimum vitreal values of R_{Tot} were calculated over model outputs from the interval $t \in [9,12]$ weeks. Circles show the maximum/mean/minimum values of R_{Tot} for the default parameter set (see Table 4).

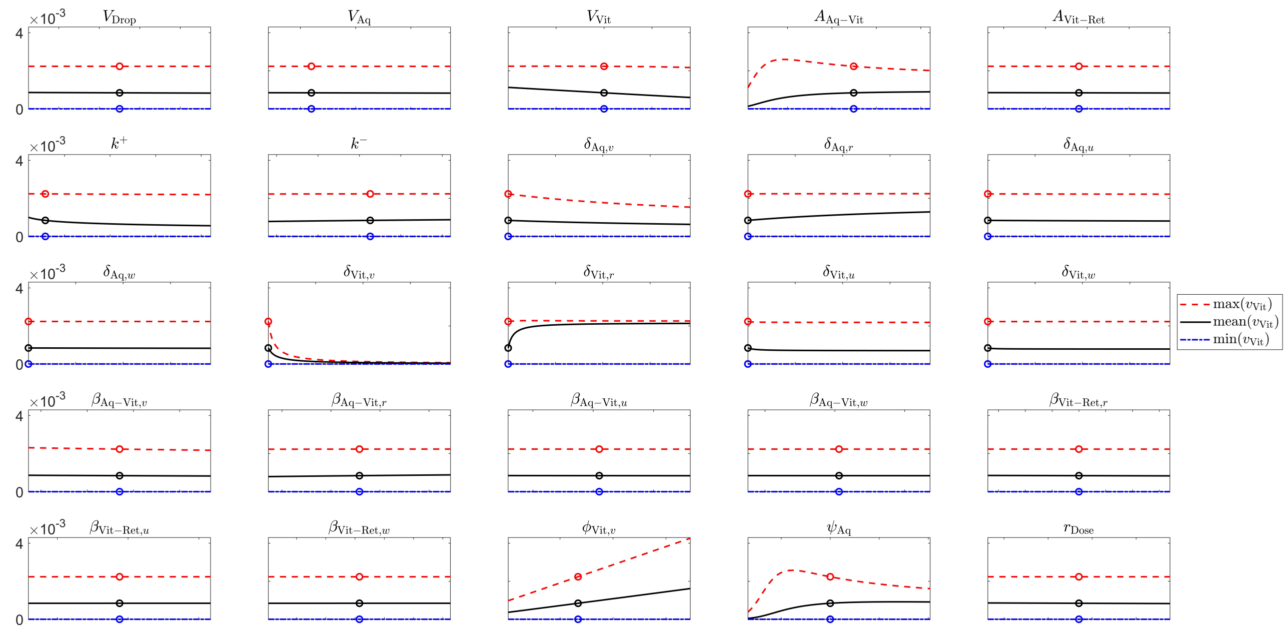

Figure S6: Sensitivity of vitreal VEGF concentration to parameter variation, with intravitreal injection therapy. Panels show variation in the maximum/mean/minimum vitreal VEGF (V) concentration, in response to variation in model parameters over biologically realistic ranges. Equations 2–12 were solved for $t \in [0, 12]$ weeks, with intravitreal injections administered at the start of weeks 1, 5, and 9. Parameters were varied individually, across 101 values uniformly distributed over the ranges given in Table 4, the remaining parameters being held at their default values given in Table 4. For each parameter set, the maximum/mean/minimum vitreal values of V were calculated over model outputs from the interval $t \in [9, 12]$ weeks. Circles show the maximum/mean/minimum values of V for the default parameter set (see Table 4).

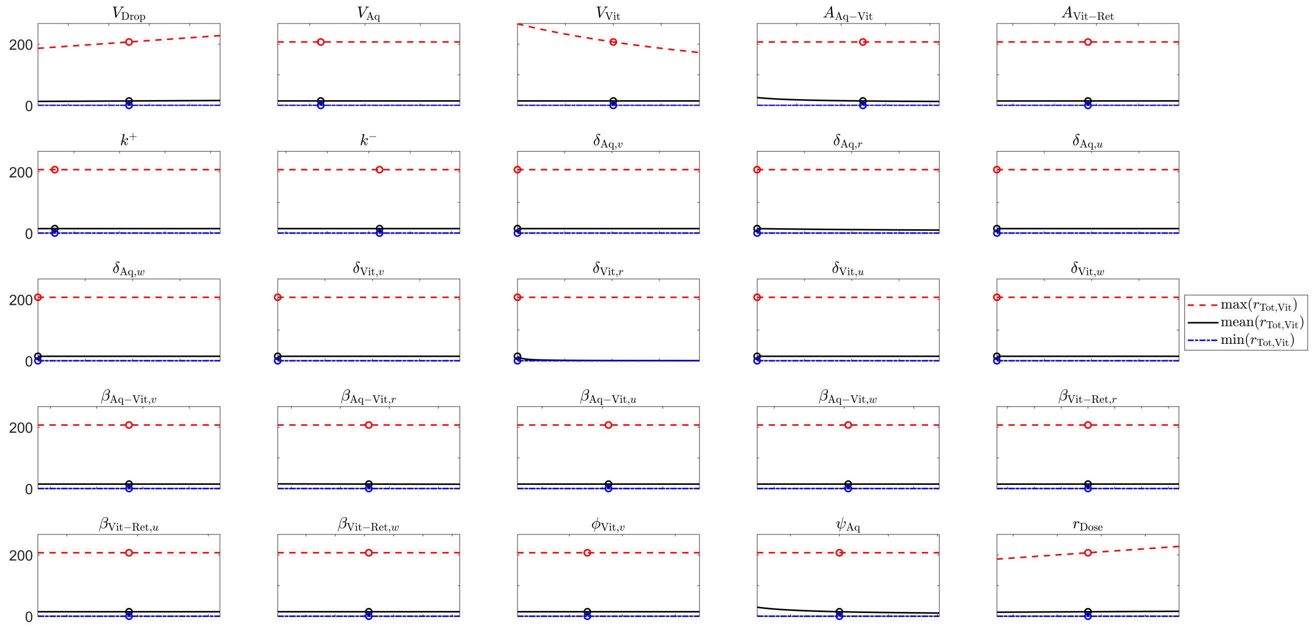

Figure S7: Sensitivity of total vitreal ranibizumab concentration to parameter variation, with intravitreal injection therapy. Panels show variation in the maximum/mean/minimum total vitreal ranibizumab (R_{Tot} = R + VR + 2RVR) concentration, in response to variation in model parameters over biologically realistic ranges. Equations 2–12 were solved for $t \in [0, 12]$ weeks, with intravitreal injections administered at the start of weeks 1, 5, and 9. Parameters were varied individually, across 101 values uniformly distributed over the ranges given in Table 4, the remaining parameters being held at their default values given in Table 4. For each parameter set, the maximum/mean/minimum vitreal values of R_{Tot} were calculated over model outputs from the interval $t \in [9, 12]$ weeks. Circles show the maximum/mean/minimum values of R_{Tot} for the default parameter set (see Table 4).
